# Progression of myopia in a natural cohort of Chinese children during COVID-19 pandemic

**DOI:** 10.1007/s00417-021-05305-x

**Published:** 2021-07-21

**Authors:** Dandan Ma, Shifei Wei, Shi-Ming Li, Xiaohui Yang, Kai Cao, Jianping Hu, Sujie Fan, Lihua Zhang, Ningli Wang

**Affiliations:** 1 Beijing Ophthalmology & Visual Sciences Key Laboratory, Beijing Institute of Ophthalmology, Beijing Tongren Eye Center, Beijing Tongren Hospital, Capital Medical University, NO.1 Dongjiaominxiang Street, Dongcheng District, Beijing, 100730 China; 2 Handan City Eye Hospital, Handan, 056000 China

**Keywords:** Myopia progression, Mydriatic spherical equivalent, COVID-19

## Abstract

**Purpose:**

To determine myopia progression in children during the COVID-19 and the related factors associated with myopia.

**Methods:**

All subjects underwent three-timepoint ocular examinations that were measured in July 2019, January, and August 2020. We compared the changes in uncorrected visual acuity (UCVA), mydriatic spherical equivalent (SE), and axial length (AL) between two periods (before and during COVID-19). A questionnaire was performed to investigate risk factors for myopia.

**Results:**

Compared with before the COVID-19, the mean (S.D.) myopia progression during the COVID-19 was significantly higher in right eyes (− 0.93 (0.65) vs. − 0.33 (0.47) D; *p* < 0.001). However, the differences in UCVA changes and the axial elongation between two periods were clinically insignificant. Through logistic regressive analysis, we found the difference of the SE changes was associated with the baseline AL (*P* = 0.028; 95% confidence interval [CI], 1.058, 2.632), online education (*P* = 0.02; 95% CI, 1.587, 8.665), and time of digital screen (*p* < 0.005; 95% CI, 1.587, 4.450).

**Conclusions:**

Children were at higher risk of myopia progression during COVID-19, which was associated with the baseline AL, the longtime online learning, and digital screen reading.


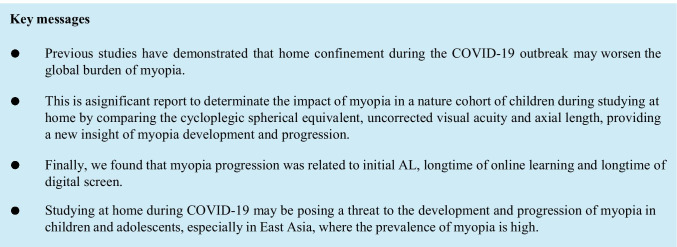


## Introduction

The novel coronavirus disease 2019 has had a global impact [1] and affected almost every aspect of people’s lives. As of January 1, 2021, more than 83.77 million have been confirmed case and 1.82 million have died globally [2]. For the first time in human history, global children could not go to school, but study at home because of the various lockdown measures imposed on populations everywhere to contain to spread of the virus. During the COVID-19 pandemic, governments around the world have provided deliver distance education at scale in an attempt to ensure continuity of learning. According to United Nations Educational, Scientific and Cultural Organization (UNESCO), as of early-September, 820 million children and youth were affected by school closures, from pre-primary to higher education. Forty-six countries were in a state of nationwide school suspension [3].

It was speculated that the closure of schools and study at home may have negative effects on children’s physical and mental health [4]. The Ministry of Education of the People’s Republic of China estimated that more than 220 million children and adolescents were kept at home and had online education in China [5]. During the COVID-19 pandemic, school was closed, but student learning was continuing online. It is necessary to investigate whether their near work increased, while outdoor activities decreased during studying at home. Students completed their learning through online learning, and the usage time of digital screen was prolonged. These effects on children may lead to the development and progression of myopia.

Myopia has become a significant public health problem. There are about 2.5 billion myopic people in the world [6]. Myopia is the most common cause of vision impairment in children [7], especially in Singapore [8], Japan [9], and China [10–13]. The international and domestic researchers were concerned about whether home confinement and studying at home during the COVID-19 pandemic worsen the global burden of myopia [14]. However, data on myopia ocular biometric parameters in children during studying at home were lacking.

From January 2020 to August 2020, schools were closed, and students completed deliver distance education at home. Ministry of Education of the People’s Republic of China said that the rate of myopia among Chinese primary and middle school students increased by 11.7% in the first 6 months of the year [15]. It is urgent to investigate the development and progression of myopia in children and its influencing factors during studying at home compared with pre-outbreak. As far as we know, this is the significant report to determinate the impact of myopia in a nature cohort of children during studying at home by comparing the mydriatic spherical equivalent, uncorrected visual acuity, and axial length, providing a new insight of myopia development and progression.

## Methods

### Studying cohort

Studied population included, children aged 8 to 10 years with the best-corrected visual acuity at least Log MAR VA 0.0 in both eyes in Handan of Hebei Province, China. Exclusion criteria were those with systemic disease or former or present eye disease or injury. Children with atropine or orthokeratology or any conditions that might have influenced myopia were excluded. A total of 208 children were examined three times, including ocular examinations and a questionnaire. Baseline data were collected in July 2019. Two follow-up visits were conducted in January 2020 and August 2020. The questionnaires were performed in August 2020.

In order to determine the change value of children’s eye parameters in the 6 months before the COVID-19 outbreak, we set a control group. We included 83 children with myopia who were admitted to Beijing Tongren Hospital, China, from April 2018 to July 2018 and followed up for 6 months. All children met the inclusion criteria.

The research was approved by the Ethics Committee of Beijing Tongren Hospital, Capital Medical University. All participating students gave written informed consent and adhered the principles of the Declaration of Helsinki.

### Procedures

Children underwent a comprehensive and standardized ocular examination, including uncorrected visual acuity, cycloplegic refraction, and axial length at baseline and two follow-up visits. In addition, a detailed questionnaire was conducted at the last follow-up.

All children were conducted for distance visual acuity (VA) without spectacles, using logarithmic visual acuity chart (Precision Vision, La Salle, IL, USA) at a distance of 4 m. The chart was retro-illuminated and has 70 tumbling “E” optotypes with five letters on each line. Children were examined monocularly (left eye followed by right eye); the detailed procedure has described elsewhere [16].

The autorefractor-keratometry (KR8800, Topcon, Tokyo, Japan) was used to measure after cycloplegic autorefraction. For each student, three drops of 1% cyclopentolate (Cyclogyl, Alcon-Convreur, Rijksweg, Belgium) were administered at interval of 5 min apart. If the pupil size was less than 6.0 mm after 30 min, a fourth drop of 1% cyclopentolate was administered [17]. Three auto-refraction readings were taken, and the average was recorded.

The axial length was measured by the IOL master (Carl Zeiss Meditec AG, Jena, Germany). Five repeated measurements were detected and obtained the average AL value before cycloplegic.

The questionnaire used in our study was derived from Anyang Children Eye Study, and the detailed procedure has been described elsewhere [18]. To control interview bias, the questionnaire survey was performed a pilot study on the validity and reliability [12]. Generally, the questionnaire mainly focusses to collect subjects near work and outdoor activity-related parameters, including time (hours/day) and modes of near work and outdoor activities before and during studying at home. Near work included homework, reading books, painting, playing chess, using computer, and using mobile phone. Outdoor activities included bicycle riding, running, swimming, and playing football. The parents and children were asked the time (hours/day) and parameter of online education during study at home. All questionnaires were completed by children and parents at the same time.

### Definitions

The spherical equivalent (SE) is calculated according to the standard formula of the algebraic sum of the dioptric powers of the sphere and half of the cylinder (sphere + 0.5 × cylinder). Myopia was defined as SE ≤  − 0.5D [19]. The emmetrope and hyperopia were defined as SE between − 0.5D and + 0.5D, and great than + 0.5D, respectively [20].

In period one (July 2019 to January 2020), the changes of subjects’ refractive error were the changes within 7 months before outbreak, and we set them as the control period. In period two (January 2020 to August 2020), the changes of refractive error were the changes within 7 months during the COVID-19 pandemic study at home, and we set them as the experimental period. The difference of the SE’s change between the control period and experimental period was defined as the change of SE in the period one minus the change of SE in the period two. The value of difference of the SE’s change was classified as flat (SE ≥ 0D) and decline (SE < 0).

### Data management and statistical analysis

The database used Epidata software 3.1 (The Epidata Association, Odense, Denmark), and statistical analysis was performed using SPSS 26.0 (IBM SPSS Statistics, Chicago, IL).

Baseline variable of ocular characteristics is described by maximum, minimum, mean, and standard deviation. Nonparametric tests were used to investigate statistical significance among control group, period one, and period two. Paired t test that is used to conform to normal distribution and rank sum test that is used for non-normal distribution were used to determine statistical significance between the period one and period two. To detect potential risk factors of development of myopia, the binary logistic regression was used to analyze the influencing factors of eye parameters. Since large correlation coefficients for cycloplegic SE were observed between the two eyes (*r* = 0.73, *P* < 0.001), only data from right eyes were included in the analyses. A two-sided *P*-values less than 0.05 were considered statistically significant.

## Results

A total 208 children completed examinations and were included for analysis. A total of 109 (52.4%) subjects were male. The age range was 8–10 years old with a mean age of 8.9 ± 0.69 years. Among the 208 children included in the study, 90 were myopia, 77 were emmetropia, and 41 were hyperopia. Ocular characteristics at baseline are presented in Table 1. The mean uncorrected visual acuity of right eyes was 0.19 ± 0.22. The mean axial length and spherical equivalent of right eyes were 23.08 ± 0.92 mm and − 0.50 ± 1.25 D.

**Table 1:** The value of ocular characteristics of right eyes at baseline

	Maximum	Minimum	Mean	Standard deviation
UCVA	1.00	− 0.10	0.17	0.22
Axial length, mm	25.60	20.05	23.08	0.91
Spherical equivalent, D	1.75	− 7.88	− 0.50	1.25

Among the 83 children in the control group, 38 children were myopia, 25 children were emmetropia, and 20 children were hyperopia. The mean axial length was 23.85 ± 0.94 mm in the control group and 23.08 ± 0.91 mm in the study group. There was no statistical difference in axial length between the two groups (*P* > 0.05). The mean SE of the control group and the study group were − 0.47 ± 1.38 D and − 0.50 ± 1.25 D, respectively (*P* > 0.05).

During the 7 months before the COVID-19 outbreak, in the study group, there was no significant difference in the SE change of myopia, emmetropia, and hyperopia. The mean change of hypermetropic children was − 0.38 ± 0.80 D, and the mean change of emmetropia and myopia was − 0.66 ± 0.83 D and − 0.63 ± 0.90 D (*P* = 0.217). In January 2020, 122 out of 208 children were myopia. During the 7 months after COVID-19 outbreak, 178 children were myopia. No variation toward less myopia or more hyperopia was found.

**Fig. 1:**
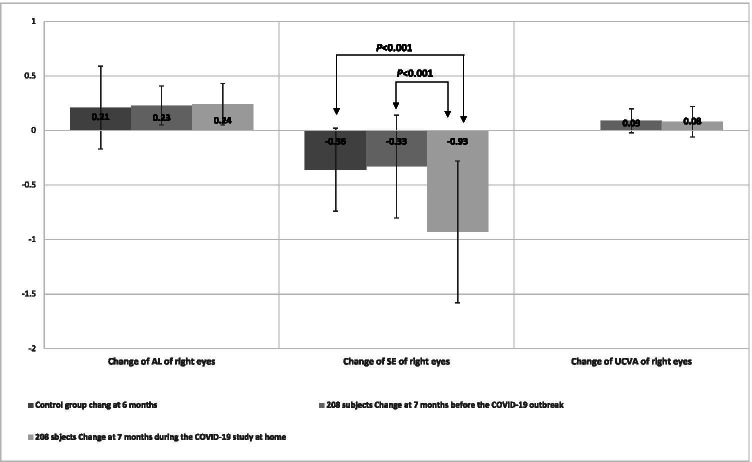
The changes of ocular parameters among control group, before and during COVID-19 pandemic study at home

Figure 1 shows the changes of ocular parameters before and during studying at home. The SE changes of right eyes were 0.60D (*P* < 0.001). The SE changes between the control period and during studying at home were significantly different (*P* < 0.001). The changes of UCVA between two periods in right eyes were not significantly different (*P* > 0.05). Over the 7 months before outbreak, the mean value of axial elongation was 0.23 ± 0.18 mm in the right eye, which was not significantly difference than 0.24 ± 0.19 mm during outbreak (*P* = 0.37).

Before the COVID-19 outbreak, in the study group, there was no statistically significant difference in the amount of time spent on various activities by myopic, emmetropic, and hyperopic children. The time spent on near work was 3.06 ± 1.98 h/day in children with hyperopia, 3.45 ± 2.82 h in children with emmetropia, and 3.32 h in children with myopia (*P* = 0.640). Children with myopia, emmetropia, and hyperopia spent 2.06 ± 2.065 h/day, 1.90 ± 1.36 h/day, and 1.83 ± 2.03 h/day on outdoor activities, respectively (*P* = 0.348).

**Fig. 2:**
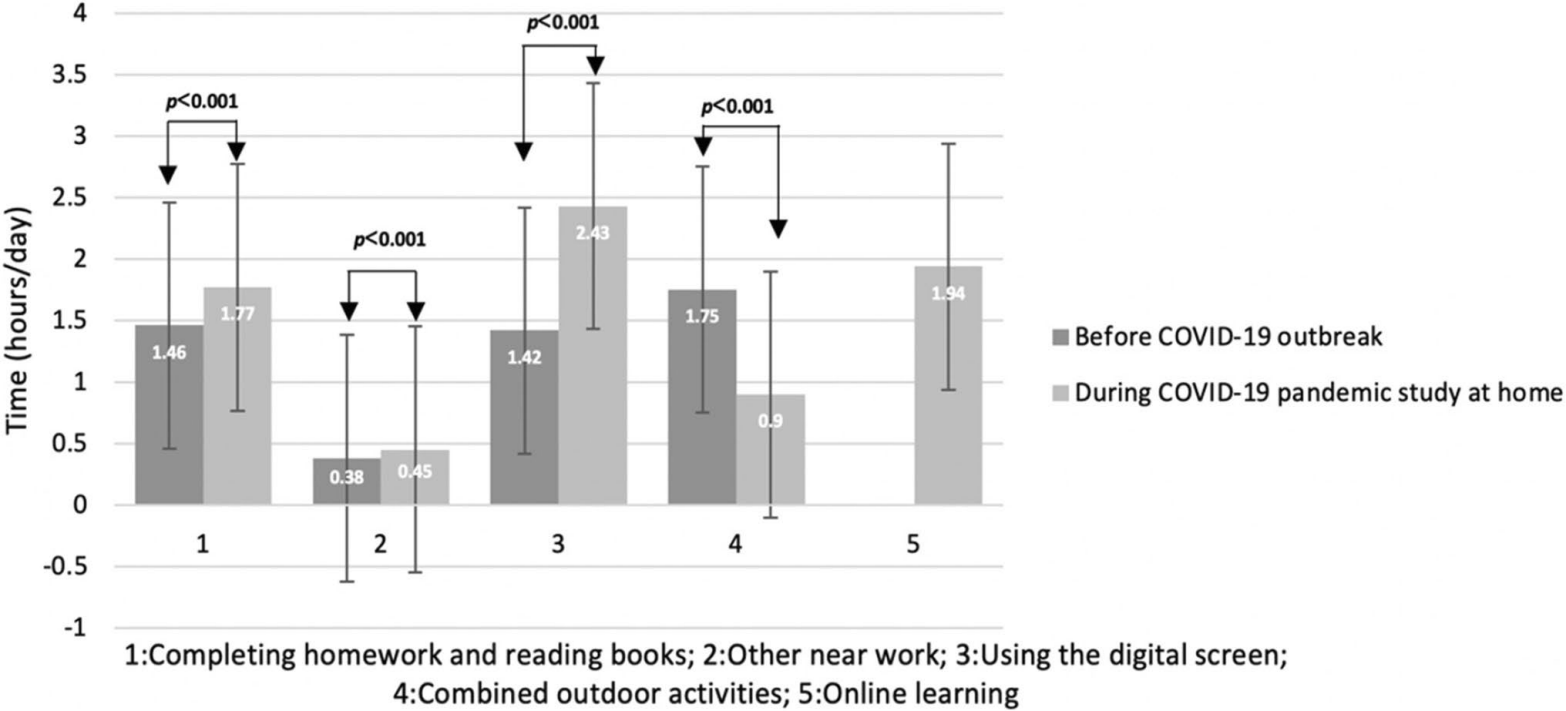
Time spent in near work and outdoor activities (hours/day) before and during studying at home

Compared with before COVID-19 outbreak, during studying at home, time spent in homework, and reading books increased from 1.46 ± 0.98 to 1.77 ± 1.06 h/day. Time spent in other near work like painting and playing chess before and during COVID-19 pandemic were 0.38 ± 0.47 h/day and 0.45 ± 0.6 h/day, respectively. Before COVID-19 outbreak, children spent 1.42 ± 1.77 h/day in digital screen included using computer and mobile phone, with no online education. During COVID-19 outbreak, children spent 2.43 ± 2.19 h/day in digital screen, and 1.94 ± 1.05 h/day in online education. Compared with before COVID-19 outbreak, during studying at home, outdoor activities decreased from 1.75 ± 1.52 to 0.90 ± 1.04 h/day. The details are presented in Fig. 2.

**Fig. 3:**
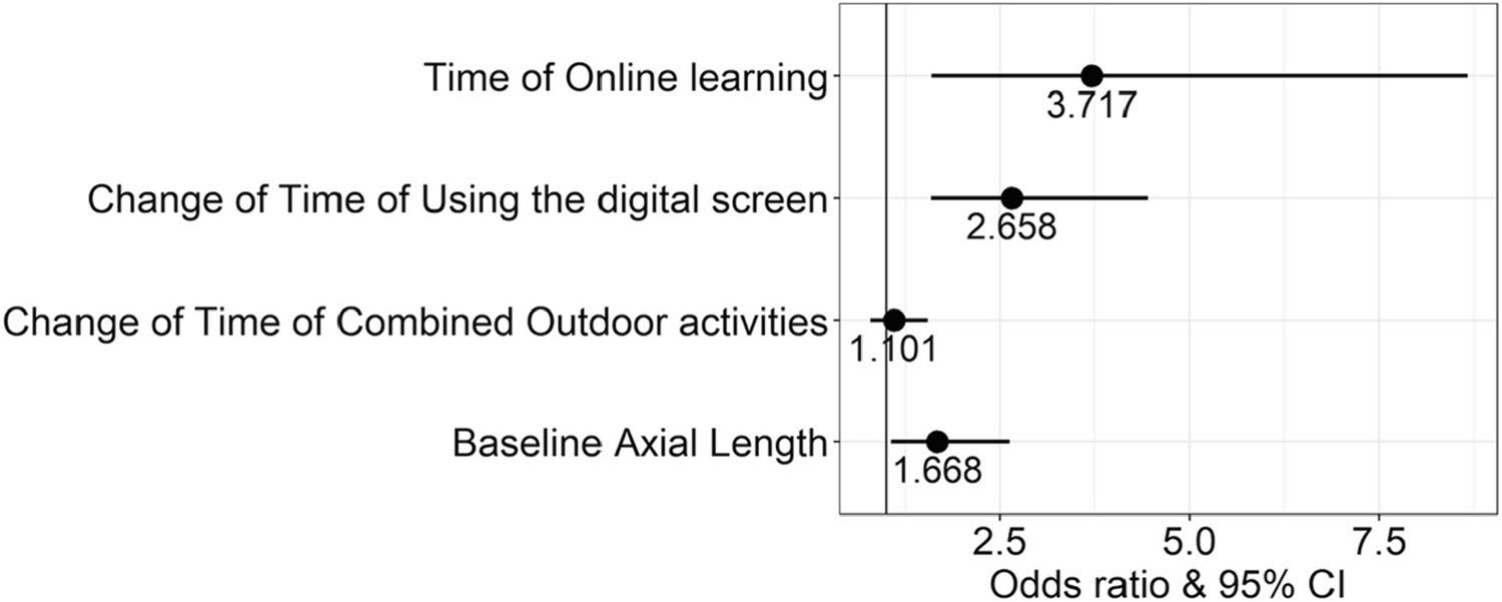
Logistic regression analysis of the difference of the SE’s change

A binary logistic regression was employed to analyze the relations of the different factors associated with myopia, including the age, gender, baseline UCVA, baseline AL, baseline SE, and the changes of time between before and after homework and reading, digital screen, other near work, as well as the change of time of online learning during the COVID-19, and the change of time of outdoor activities. The results showed that the difference of the SE’s change was related to the baseline AL, the change of time of using digital screen, and the time of online learning (Fig. 3).

## Discussion

It was reported that the rate of myopia among Chinese primary and middle school students increased by 11.7% in the first 6 months of the year [15]. However, data on myopia progression and mydriatic spherical equivalent in children during studying at home were lacking. As far as we know, this is the significant report analyzing the progression of myopia in children during studying at home. The mean SE change was − 0.9D in 7 months during studying at home. However, in the 7 months before the COVID-19, the mean myopia progression was − 0.3D and this difference was found to be statistically significant. Moreover, it was found that the difference of the mydriatic spherical equivalent changes was related to the children’s baseline AL, longtime online leaning, and digital screen reading.

The control group about myopia progression in Chinese children reported that at the 6-month follow-up, the mean values of myopia progression was − 0.36D, and mean value of axial elongation was 0.21 mm [21]. These results were similar as ocular parameter’s change before the COVID-19 outbreak. Compared with the control group, the average progression of SE during 7-month study at home was statistically significant (Fig. 2).

A summary of the myopia progression in previous studies is shown in Table 2. Our study found the myopia progression of − 0.3 ± 0.5 D in 7 months before the COVID-19 what was similar to the previous studies of Chinese and foreign children showing myopia progression of − 0.3D in 6 months or − 0.6D in a year [22–28]. There were also some studies presenting higher myopia progression [25, 29–31]. We believe that this may be related to different genetic and environmental factors as well as differences in age and the size of refractive error in different studies. Compared with previous studies, we found that myopia progressed rapidly during studying at home [22–32]. The myopia progression was − 0.9D in 7 months during studying at home.

**Table 2:** Key results and myopia progression from previous studies

Reference	Location	Age, y	Sample size	Duration, months	Average myopia, D	Myopia progression, D
Donovan L et al., 2012[22]	China	6–12	85	6	NA	− 0.31 ± 0.25 for summer − 0.53 ± 0.29 for winter
Fujiwara M et al., 2012[24]	Japan	10–13	92	6	− 4.40 ± 1.38	− 0.35 ± 0.04 for summer − 0.28 ± 0.06 for winter
Cui D et al., 2013[32]	Denmark	8–14	235	6	− 2.24 ± 1.39	− 0.287 ± 0.266
Jane G et al., 2014[26]	USA	6–12	469	6	− 2.54 ± 0.84	− 0.35 ± 0.34 for winter − 0.14 ± 0.32 for summer
Yu S et al., 2015[29]	China	6–15	900	6	NA	− 0.56 ± 0.37
Clark et al., 2015[27]	USA	6–15	NA	12	− 2.0 ± 1.5	− 0.6 ± 0.4
Yam et al., 2018[30]	Hong Kong	4–12	109	12	− 3.85 ± 1.95	− 0.81 ± 0.53
Pei w, et al., 2018[31]	Taiwan	6–7	41	12	NA	− 0.79 ± 0.38
Sacchi et al., 2019[25]	Italy	5–16	50	12	− 2.63 ± 2.68	− 1.09 ± 0.64
Larkin et al., 2019[28]	USA	6–15	98	12	− 2.8 ± 1.6	− 0.6 ± 0.4
Yun c et al., 2020[23]	China	8–15	144	12	− 3.16 ± 1.13	− 0.61 ± 0.31

Our study showed that compared with before the COVID-19, there was a significant change in SE, but no significant change in axial length and UCVA during the COVID-19 pandemic. We speculate that it may be transient myopia caused by accommodative spasm. More data and studies are needed to support this hypothesis in the future.

A large sample study of Chinese children found that myopia was significantly related to near work like continuous reading [33]. Near work was considered as a risk factors for myopia through increased accommodative demand [33]. Longtime near work causes the increase of prevalence and incidence of myopia in children [34, 35]. Long-term usage of electronic screens, near work, and limited outdoor activities were associated with the onset and progression of myopia [36].

Digital screen time has been reported as a risk factor that could increase myopia [37]. During the COVID-19 pandemic, students were away from the school classroom and have the online education that greatly increased the digital screen time.

Due to kept at home, children and adolescents spent more time reading books, watching TV, playing video games, and using computers, tablets, and smartphones. During the COVID-19 pandemic, Canadians aged 15–49 spent 66% more time watching TV and 35% more playing video games than before the COVID-19 outbreak [38]. In South Korea, 79 (81%) of 97 parents said their children’s digital time had increased [39].

Previous studies have found that outdoor time has a protective effect on myopia onset [39–41]. During COVID-19 pandemic, the outdoor activities of children and teenagers have been reduced because of the various lockdown measures imposed on populations everywhere to contain to spread of the virus. Although the outdoors activities time decreased, our study did not show any relation between outdoor activity and myopia progression. We speculated that it could be due to the small sample size of this study that the results were different from those of previous studies.

Although it is the unique study investigating the COVID-19 lifestyle impact on myopia development and progression in natural cohort of children, this study has several limitations. First is the selection bias due to a relatively small sample size. Secondly, the data on time and parameters of near work and outdoor activities were not directly measured, but were acquired by questionnaires, which may have led to recall bias. To minimize this bias, all questionnaires were completed by children and parents. Thirdly, previous studies [22, 26] found myopia progressed more slowly in summer than in winter. Handan has seasonal changes through the year. In our study, the two periods of observation were in different seasons, which may bias the outcomes. However, home isolation during the COVID-19 epidemic mainly occurred in the summer months, and our study found the myopia progression was greater in children during this period. This indicates that changes in living styles and learning environment due to the COVID-19 lead to greater myopia progression.

## Conclusion

In summary, the nature cohort study in children revealed that the children were at risk of myopia progression during the COVID-19 pandemic study at home than 7 months before the outbreak. The myopia progression was related to the initial AL, the longtime of online learning, and the longtime of digital screen. Studying at home during COVID-19 may be posing a threat to the development and progression of myopia in children and adolescents, especially in East Asia, where the prevalence of myopia is high.

## Data Availability

All data generated or analyzed during this study are included in this published article.
